# Therapeutic potential of Triptolide in inhibiting breast cancer-induced bone destruction – PTHrP as a therapeutic target

**DOI:** 10.3389/fphar.2025.1512631

**Published:** 2025-05-15

**Authors:** Wu Di, Li Minghan, Huang Zining, Zhou Chunshi, Hu Xiangyu, Wang Zidong, Wu Gang

**Affiliations:** ^1^ Hubei Provincial Hospital of Traditional Chinese Medicine, Affiliated Hospital of Hubei University of Chinese Medicine, Wuhan, Hubei, China; ^2^ Hubei University of Chinese Medicine, Wuhan, Hubei, China; ^3^ School of Life Sciences, Hubei University, Wuhan, Hubei, China; ^4^ School of Sports Medicine, Wuhan Institute of Physical Education, Wuhan, Hubei, China

**Keywords:** Triptolide, osteoclast, osteoblast, bone metastasis, breast cancer, PTHrP

## Abstract

**Introduction:**

Bone metastases are a common and severe complication in advanced breast cancer, affecting approximately 65% to 70% of patients and significantly reducing survival time. Osteolytic bone metastases, in particular, are challenging to manage due to their association with skeletal-related events (SREs) that accelerate disease progression and diminish the quality of life. These metastases are driven by a complex interaction between breast cancer cells and the bone microenvironment, leading to increased osteoclast activity and bone destruction. Current treatments, such as bisphosphonates, primarily aim to inhibit osteoclast function but are associated with serious side effects, underscoring the need for alternative therapies. Triptolide (TP), a bioactive compound derived from the traditional Chinese medicinal herb Tripterygium wilfordii Hook F. (TwHF), has demonstrated potent anti-tumor and anti-inflammatory properties, especially in abnormal bone remodeling disorders. This study aims to investigate the therapeutic potential of TP in treating breast cancer-induced bone metastases by examining its effects on osteoclastogenesis and tumor–bone microenvironment interactions.

**Methods:**

Mouse bone marrow cells, RAW264.7 pre-osteoclasts and MC3T3‐E1 pre-osteoblasts were cultured with MDA‐MB-231 breast cancer cells or their conditioned medium to replicate the tumor microenvironment. Osteoclast formation was assessed via TRAP staining. Translational and transcriptional expression of key signaling molecules and related markers were determined using western blot and RT‐PCR. Binding interactions between TP and parathyroid hormone-related protein (PTHrP) were analyzed using microscale thermophoresis and molecular docking.

**Results:**

TP treatment significantly reduced osteoclastogenesis in both co‐culture and conditioned medium systems. Our findings suggest that TP inhibits NF-κB and ERK signaling pathways, reduces breast cancer-induced osteoclastogenesis, and decreases NFATc1, CTSK, and RANKL expression. Molecular assays revealed a direct binding affinity between TP and PTHrP, suggesting TP interferes with PTHrP‐mediated signaling that promotes osteoclast activity.

**Discussion:**

This study demonstrates that Triptolide effectively inhibits breast cancer‐induced osteolytic bone metastasis by suppressing key osteoclastogenic signaling pathways and modulating the tumor-bone microenvironment. We provide the first evidence of a direct interaction between TP and PTHrP, suggesting a novel mechanism through which TP may disrupt PTHrP-mediated osteoclast activation. These findings position TP as a promising alternative to current anti‐resorptive therapies for managing breast cancer-associated bone metastases.

## 1 Introduction

Bone metastases are a common and significant complication in advanced breast cancer, affecting approximately 65%–70% of patients, with a median survival time ranging from 19 to 25 months (Wang et al., 2020). These metastases are particularly challenging to manage, as the development of skeletal-related events (S.R.E.s) can accelerate disease progression and severely impact the patient’s quality of life (Coleman et al., 2020). Histologically, bone metastases are classified into three main types: osteolytic, osteogenic, and mixed. This study focuses specifically on osteolytic bone metastases, which are most associated with breast cancer. The pathophysiology of osteolytic metastases is complex and is best understood through the “Seed and Soil” hypothesis, which suggests that tumor cells enter the circulatory system, disseminate throughout the body, and colonize specific tissues, including bone (Paget, 1989). In the bone microenvironment, osteoclast-mediated bone resorption creates a niche conducive to tumor cell survival and early metastatic formation. Factors released during bone resorption, such as transforming growth factor-beta (TGF-β), can awaken dormant tumor cells and promote their proliferation (Nguyen et al., 2009). Additionally, parathyroid hormone-related protein (PTHrP), synthesized by tumor cells, disrupts leukemia inhibitory factor receptor (LIFR) signaling, further facilitating tumor cell exit from dormancy and enhancing metastatic growth (Johnson et al., 2016).

The interaction between breast cancer cells and bone cells initiates a vicious cycle, where the signals from cancer cells promote both tumor progression and bone destruction. Notch ligand Jagged1, expressed by breast cancer cells, stimulates the release of interleukin-6 (IL-6) from osteoblasts, which in turn promotes tumor growth and osteoclast activation (Sethi et al., 2011). Breast cancer cells can directly stimulate osteoclastogenesis by secreting factors such as receptor activator of nuclear factor kappa-Β ligand (RANKL), IL-1, IL-6, and tumor necrosis factor-alpha (TNF-α) (Le Pape et al., 2016). They can also indirectly promote osteoclastogenesis by inducing the bone matrix to produce more RANKL via cytokines such as platelet-derived growth factor (PDGF), urokinase-type plasminogen activator (uPA), and PTHrP. Current treatments for bone metastases control disease by inhibiting osteoclast activity but often associated with serious side effects (Chen et al., 2010; Orozco and Maalouf, 2012), therefore highlight the urgent need for safer alternative therapies.

One potential alternative treatment is the traditional Chinese medicine “Lei Gong Teng” (Tripterygium wilfordii Hook F., or TwHF), which has a long history of use in China for treating bone and joint-related diseases. Triptolide (TP), a bioactive compound derived from TwHF, has been extensively studied for its anti-tumor and anti-inflammatory properties (Zhou et al., 2012). Although the exact mechanisms of TP are not fully elucidated, it has demonstrated efficacy in treating conditions characterized by disrupted bone remodeling, such as rheumatoid arthritis and osteosarcoma (Zhao et al., 2016; Fan et al., 2018). These conditions share the feature of overactive osteoclast activity, suggesting that TP may similarly target osteoclastogenesis through the inhibiting key signaling pathways such as NF-κB, ERK, PI3K-Akt, and MAPK. TP has been shown to downregulate RANKL expression while enhancing OPG production, thereby reducing the RANKL/OPG ratio and suppressing osteoclast differentiation. This mechanism has been observed both *in vitro* and in various animal models of bone loss and inflammation (Cui et al., 2020; Yang et al., 2019). Li and Zhang further confirmed that TP suppresses IL-1β-induced activation of the RANK/RANKL/OPG axis in osteoarthritic rats, highlighting its dual anti-inflammatory and anti-osteoclastogenic action in degenerative joint disease (Li and Zhang, 2020). In a comprehensive review, we recently summarized the therapeutic effects of TP in various bone-related disorders, highlighting its anti-resorptive potential through suppression of RANKL-mediated signaling and its translational relevance in osteolytic diseases such as rheumatoid arthritis and bone metastasis (Gang et al., 2022).

Given that abnormal osteoclast activity is a hallmark of bone metastases in breast cancer, we hypothesize that TP could inhibit this activity within the tumor microenvironment, thereby mitigating bone destruction associated with breast cancer metastases. Our findings demonstrate that TP effectively inhibits the activation of NF-κB and ERK signaling pathways, reducing breast cancer cell-induced osteoclastogenesis. Moreover, TP was found to decrease the expression of RANKL in osteoblasts, which is modulated by the tumor microenvironment, thus indirectly inhibiting abnormal osteoclast activity. PTHrP is a well-known regulator of osteoclastogenesis, particularly in the context of cancer-induced bone disease. It acts through the PTH/PTHrP receptor to increase RANKL expression while suppressing OPG in osteoblasts promoting osteoclast differentiation and activity, leading to bone resorption. Recent studies have expanded our understanding of PTHrP importance in the bone biology. Fragments, like PTHrP(12-48), have been shown to suppress osteoclast formation and promote osteoclast apoptosis (Kamalakar et al., 2017), while others, like full-length PTHrP, promote osteolysis in skeletal malignancies (Frieling et al., 2017). Additionally, tumor-derived PTHrP drives bone degradation through RANKL-mediated osteoclast activation in cancer-associated bone loss (Dougall and Chaisson, 2006). The link between TP and PTHrP in osteoclastogenesis remains poorly characterized.

Therefore, here in this study, we provide evidence for the interaction of PTHrP and TP which could indirectly influence PTHrP-mediated pathways by reducing the signaling cascades that promote RANKL expression and osteoclastogenesis.

In nutshell, our study focuses on tumor-induced osteoclastogenesis, offering a more clinically relevant model than traditional cytokine- or RANKL-based systems. By employing co-culture and conditioned medium approaches, it effectively simulates the breast cancer–bone microenvironment, while uncovering both direct and indirect mechanisms through which Triptolide modulates osteoclast activity. Also, our study is the first to provide evidence of a novel interaction between TP and PTHrP.

## 2 Materials and methods

### 2.1 Cell lines and reagents

MDA-MB-231, RAW264.7, and MC3T3-E1 cells were procured from Procell Co., Ltd., (Wuhan, China) and maintained as per the instruction. Supplementary Table S1 lists details of all the reagents.

### 2.2 Isolation of murine bone marrow cells (BMCs)

To determine whether TP inhibits osteoclast differentiation, mouse BMCs undergoing osteoclast differentiation in response to RANKL were treated with or without TP. All experimental animals were housed at the Hubei Provincial Food and Drug Safety Evaluation Center and handled in accordance with Chinese guidelines for ethical review of laboratory animal welfare (This experiment was reviewed and approved by the Hubei Provincial Food and Drug Safety Evaluation Center, Approval No. 20231–20246). Tibiae and femurs were aseptically removed from 8-week-old female BALB/c mice (procured from the Wuhan moubaili biotechnology CO., Ltd.). The bone ends were excised using scissors, and the marrow cavities were flushed with 1 mL of α-MEM using a sterile 27-gauge needle. The bone marrow cells were then washed once with α-MEM and resuspended in α-MEM supplemented with 10% fetal bovine serum (FBS) and 10 µg/mL RANKL. The cells were cultured at a density of 1 × 10^5^ cells/0.5 mL per well in 24-well plates. After 3 days, the culture medium was replaced, allowing the bone marrow cells to be utilized as osteoclast precursor cells.

### 2.3 Cell viability assay

The cells were plated in 96 well plate at a density of 5 × 10^3^ cells/well and allowed to attach for overnight. Subsequently, cell medium was removed and replaced with fresh DMEM containing various concentrations of TP. Cell proliferation was measured at 24, 48, or 96 h using the Cell Counting Kit-8 according to manufacturer’s instructions. Briefly, 10 μL of CCK-8 reagent was added into each well and incubated at 37°C in a 5% CO2 incubator for 2 h. The optical density (OD) of formazan dye produced was measured by Multiskan EX ELISA reader at 450 nm. Each cell proliferation assay was performed in triplicate.

### 2.4 Conditioned medium

Conditioned medium from MDA-MB-231 cells was used to simulate the tumor-derived soluble factors that influence osteoblast and osteoclast activity. This approach allows for the study of tumor–bone signaling in a controlled manner, especially when investigating paracrine effects. For this, MDA-MB-231 cells were seeded at 5 × 10^4^ cells/cm^2^. After 48 h, conditioned medium (CM) was collected and centrifuged at 550 g for 10 min. The supernatant was filtered using 0.2 μm filter, aliquoted, and stored at −80°C.

### 2.5 RT-PCR

Total RNA samples from RAW264.7 cells were extracted using TRIzol reagent according to the manufacturer’s protocol. cDNA was synthesized from the total RNA using PrimeScript™ RT Master Mix, followed by real-time PCR using SYBR^®^ Premix Ex Taq™ II according to the manufacturer’s instructions. All analysis was performed using an ABI 7500Real Time PCR System. Relative gene expression was calculated using the standard 2-ΔΔCq method (Livak and Schmittgen, 2001). Data are presented as the fold-change in gene expression normalized to the level of *β*-actin. The primers used for the amplification of RANKL and actin genes were with the following sequences:

• RANKL:
  • Forward primer: 5′-TCG​GGA​AGC​GTA​CCT​ACA​GAC​TAT​C-3′
  • Reverse primer: 5′-GGA​AGG​GTT​GGA​CAC​CTG​AAT​GC-3′
• Actin:
  • Forward primer: 5′-GCC​ACC​AGT​TCG​CCA​TGG​AT-3′
  • Reverse primer: 5′-GCT​TTG​CAC​ATG​CCG​GAG​C-3′

### 2.6 Western blot

Whole cell lysates were prepared in cold radioimmunoprecipitation assay (RIPA) lysis buffer containing protease and phosphatase inhibitors, equal amount of proteins were resolved and blotted as described previously (Hu et al., 2021). The signal was detected using an ECL system (Cell Signaling) and GAPDH was used as a loading control. Quantification was performed via densitometric analysis using ImageJ software (NIH).

### 2.7 Co-culture assay

In this study, we aimed to investigate the effect of TP on tumor-induced osteoclast formation in the bone environment using a co-culture model of either mouse bone marrow cells (BMCs) or murine macrophages (RAW264.7) with MDA-MB-231 breast cancer cells. The BMCs obtained from the mouse tibia were cultured with 10 ng/mL of RANKL until most cells adhered, which took about 3 days. After 3 days, the BMCs were co-cultured with MDA-MB-231 cells for direct contact to induce mature osteoblasts for another 3 days in RANKL-free media with and without TP. On day 7, TRAP staining and osteoclast counting were performed. Similarly, RAW264.7 cells were incubated for 6 days using 50% CM (10%FBS) and 50% α-MEM(10%FBS) with or without TP. The medium was changed every other day during the incubation period, and TRAP staining was performed and the number of osteoclasts counted on day 7.

### 2.8 TRAP staining

TRAP staining was performed as described previously (Hu et al., 2021). The morphology was observed using a Leica DMI6000 B microscope (Leica Microsystems GmbH) at ×200 magnification. TRAP-positive multinucleated cells were counted in 20 fields per treatment condition. TRAP-positive multinucleated cells (MBCs) with three or more nuclei were scored. The average number of TRAP-positive multinucleated cells, determined from triplicate experiments, is presented in the results.

### 2.9 Microscale thermophoresis (MST) assay

The interaction between TP and PTHrP was assessed using a Microscale Thermophoresis (MST) assay, following the manufacturer’s instructions, RED-tris-NTA 2nd Generation and Capillaries. The assay involved two primary steps: labeling and titration. First, 50 nM of PTHrP protein was labeled with 30 μM of the fluorescent dye Cy5-NHS ester. A serial two-fold dilution of the unlabeled TP was then prepared, starting from 1 mM as the highest concentration. The labeled protein was pretested, and the fluorescence response was adjusted to a range of 200–1,000 by appropriate dilution in PBS buffer (pH 8.0). Subsequently, the labeled PTHrP was carefully mixed with the serial dilutions of TP, and the samples were loaded into standard MST capillaries. The MST parameters were set with MST Power at 20% and LED Power at 60%. Fluorescence was measured using a Monolith NT.115 instrument (Nanotemper, 201410-BR-N004), and the data were analyzed using MO. Affinity Analysis Software v2.3 (Nanotemper) For the binding of Ligand to Target, The resulting dose-response curves were fitted to a one-site binding model to extract KD values.

### 2.10 Molecular docking

The crystal structure of parathyroid hormone-related protein (PTHrP) (PDB ID: 3H3G) was obtained from the Protein Data Bank. Three-dimensional structures for Triptolide (Compound CID: 107985) were obtained from the Pubchem. Docking studies were performed using AutoDock 4.2. Automated docking was used to locate the appropriate binding orientations and conformations of various compounds. Polar hydrogens were added, and Kollman charges were assigned using AutoDock Tools (ADT). Grid maps were generated using AutoGrid with a grid box size of 90 × 90 × 90 points and a grid spacing of 0.180 Å centered on the predicted binding site of the PTHrP structure. The docking simulation was performed using the Lamarckian Genetic Algorithm with 100 runs, a population size of 300, and 2.5 million energy evaluations. Docking conformations were clustered using an RMSD threshold of 1.0 Å and ranked based on binding energy. Visualization and hydrogen bond analysis were conducted using Discovery Studio 2.5.

### 2.11 Statistical analysis

Statistical analysis was conducted using either a two-tailed unpaired Student’s t-test or ANOVA with Tukey’s *post hoc* test. The analyses were performed with a minimum of three replicates using R. Results are expressed as Mean ± SEM of at least three independent experiments. A p-value of less than 0.05 was considered statistically significant.

## 3 Results

### 3.1 Effect of triptolide on the cell viability

**FIGURE 1:**
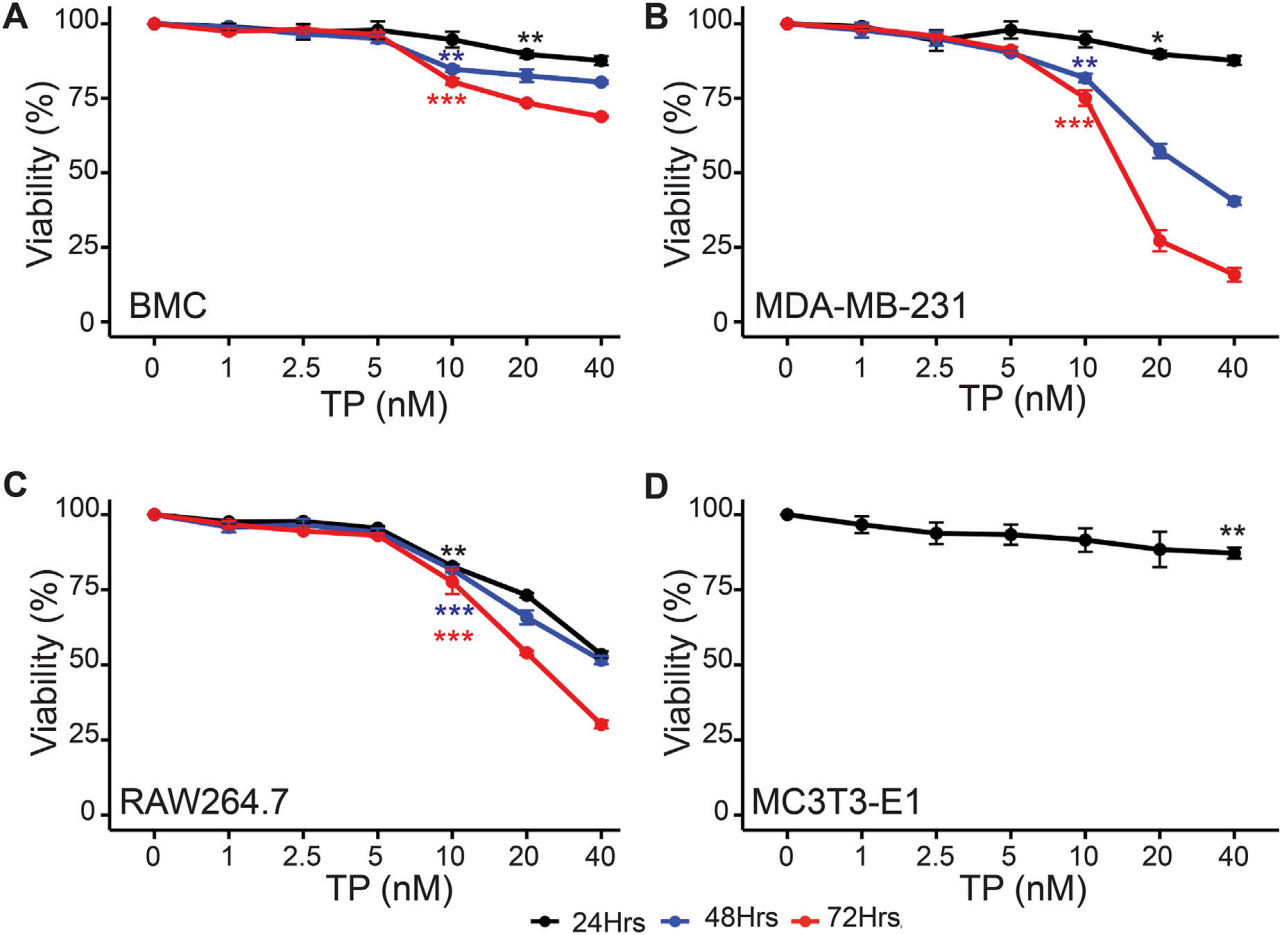
Time and Dose-Dependent Effects of Triptolide on Cell Viability. Time and dose-dependent effects of TP on viability of **(A)** Bone marrow cells, **(B)** MDA-MB-231, **(C)** RAW264.7 and **(D)** MC3T3-E1 cells (only for 24 h). Data are presented as averages of triplicate measurements with error bars representing standard error of mean. *p < 0.05, **p < 0.01 and ***p < 0.001 (Compared to no TP control).

We did not observe any significant difference in the cell viability of BMCs, MDA-MB-231 and RAW264.7 cells in the presence of TP up at a concentration of 5 nM upto 72 h (Figures 1A–C). Based on this observation, we used TP in a maximum dose of 5 nM and the experiments were carried out up to 72 h. For MC3T3-E1 cells, we did not see any adverse effect on the viability up to the concentration 10 nM at 24 h (Figure 1D). This is consistent with previous studies showing that Triptolide exhibits selective cytotoxic effects on tumor cells, while sparing non-cancerous cells under certain conditions (Huang et al., 2015; AbdulHussein et al., 2023). These observations support the usage of TP as a candidate for combinatorial therapies that aim to target cancer cells.

### 3.2 Triptolide inhibits osteoclastogenesis

**FIGURE 2:**
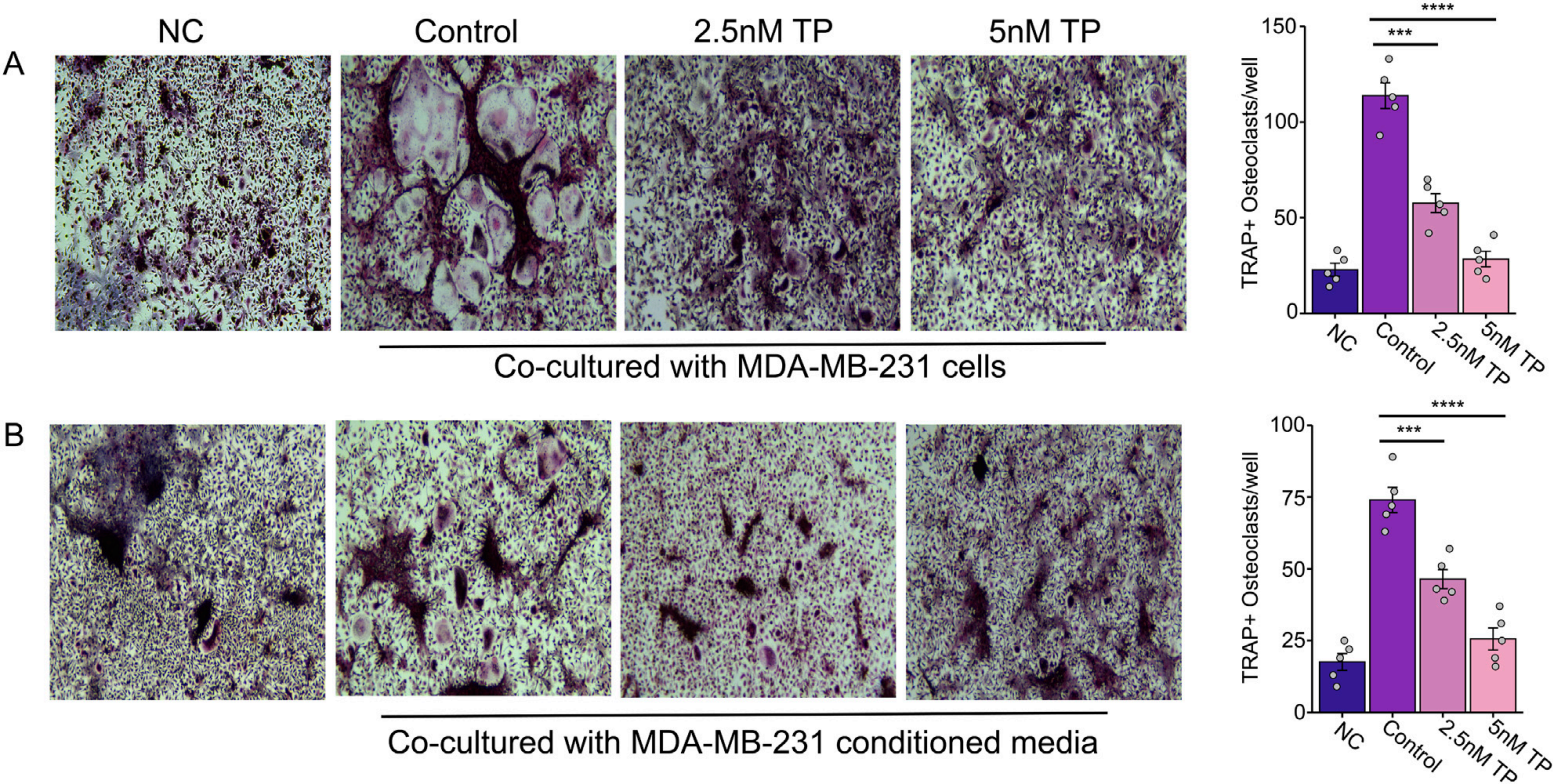
Effect of Triptolide on Osteoclastogenesis in Bone Marrow Cells Cultured with MDA-MB-231 Cells and Conditioned Media. Effect of TP on the bone marrow cells transformed osteoclasts as measured by TRAP staining when cultured with MDA-MB-231 cells **(A)** and with MDA-MB-231 condition media **(B)**. Representative images and respective quantification of multinucleate cells (×200 magnification) following TRAP staining in different conditions. Data are presented as averages of five measurements with error bars representing standard error of mean. ***p < 0.001 and ****p < 0.0001.

To determine the effect of TP on osteoclastogenesis, we co-cultured bone marrow cells (BMCs) with MDA-MB-231 cells (late-stage breast cancer tumor cells) in the presence or absence of TP. The results of the TRAP staining showed that the BMCs cultured with tumor cells transformed into large, multinucleated cells, compared to the controls. However, we observed a significantly decreased number of osteoclasts with a smaller size and fewer nuclei in the presence of TP, and this effect was dependent on the dose (Figure 2A). While this study focused on the MDA-MB-231 breast cancer cell line, which is widely used in bone metastasis models due to its strong osteotropism and PTHrP expression, it will be interesting in future studies to explore whether TP exhibits similar effects across other breast cancer subtypes. Such investigations will help validate and expand the applicability of its mechanism in diverse tumor contexts.

Previous studies have shown that breast cancer cells secrete proteins that can stimulate osteoclast formation, contributing to bone degradation (Ney et al., 2012; Thomas et al., 1999). By using BMCs and RAW264.7 cells (which can differentiate into osteoclasts), we tested whether conditioned media (CM) from breast cancer cells could induce osteoclastogenesis without traditional cytokines.

**FIGURE 3:**
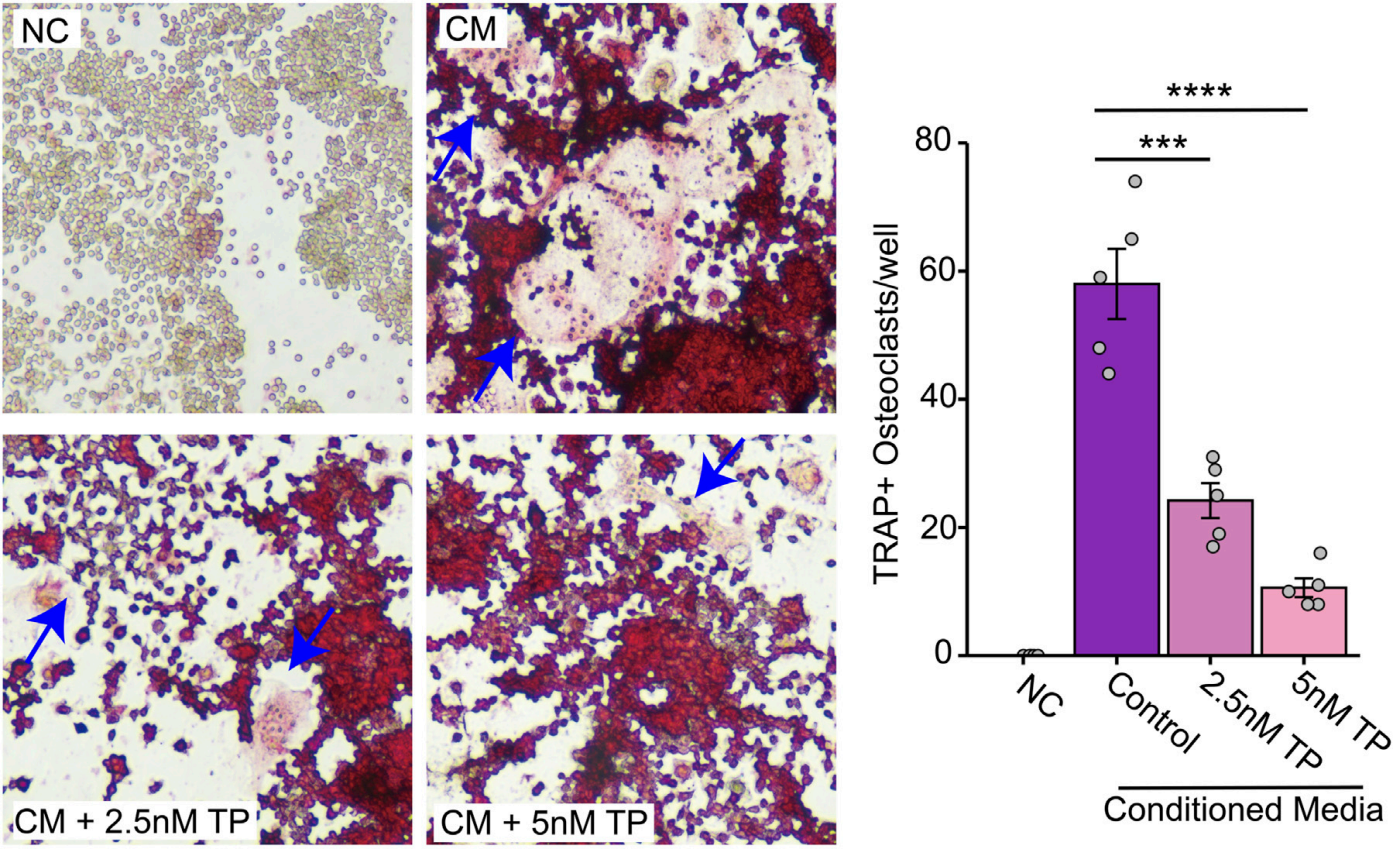
Effect of Triptolide on Osteoclast Formation in RAW264.7 Cells Cultured with MDA-MB-231 Conditioned Media. Effect of TP on the RAW264.7 cells transformed osteoclasts as measured by TRAP staining when cultured with MDA-MB-231 condition media. Representative images and respective quantification of multinucleate cells (×200 magnification) following TRAP staining in different conditions. The blue arrows indicate typical osteoclasts. Data are presented as averages of five measurements with error bars representing standard error of mean. ***p < 0.001 and ****p < 0.0001.

Similar to the direct contact experiments, we observed an increased number of osteoclast formations in the presence of the conditioned media, but this was inhibited in the presence of TP in BMCs (Figure 2B) as well as osteoclast precursor RAW264.7 cells (Figure 3). These results suggest that TP inhibits osteoclastogenesis both in direct co-culture with tumor cells and in response to soluble factors secreted by MDA-MB-231 cells.

Our results confirmed that CM from breast cancer cells indeed promoted osteoclast formation in RAW264.7 cells, even in the absence of osteoclastogenesis-related cytokines. TP significantly reduced this osteoclast formation, supporting its potential as a therapeutic agent to prevent bone degradation in breast cancer metastasis.

Therefore, we suggest that breast cancer cells can directly stimulate osteoclastogenesis through the secretion of various exogenous proteins, and that TP inhibits osteoclast formation directly stimulated by breast cancer cells (Figure 3).

### 3.3 Triptolide inhibits NF-κB signaling and ERK signaling pathways to reduce osteoclastogenesis directly stimulated by tumor cells

**FIGURE 4:**
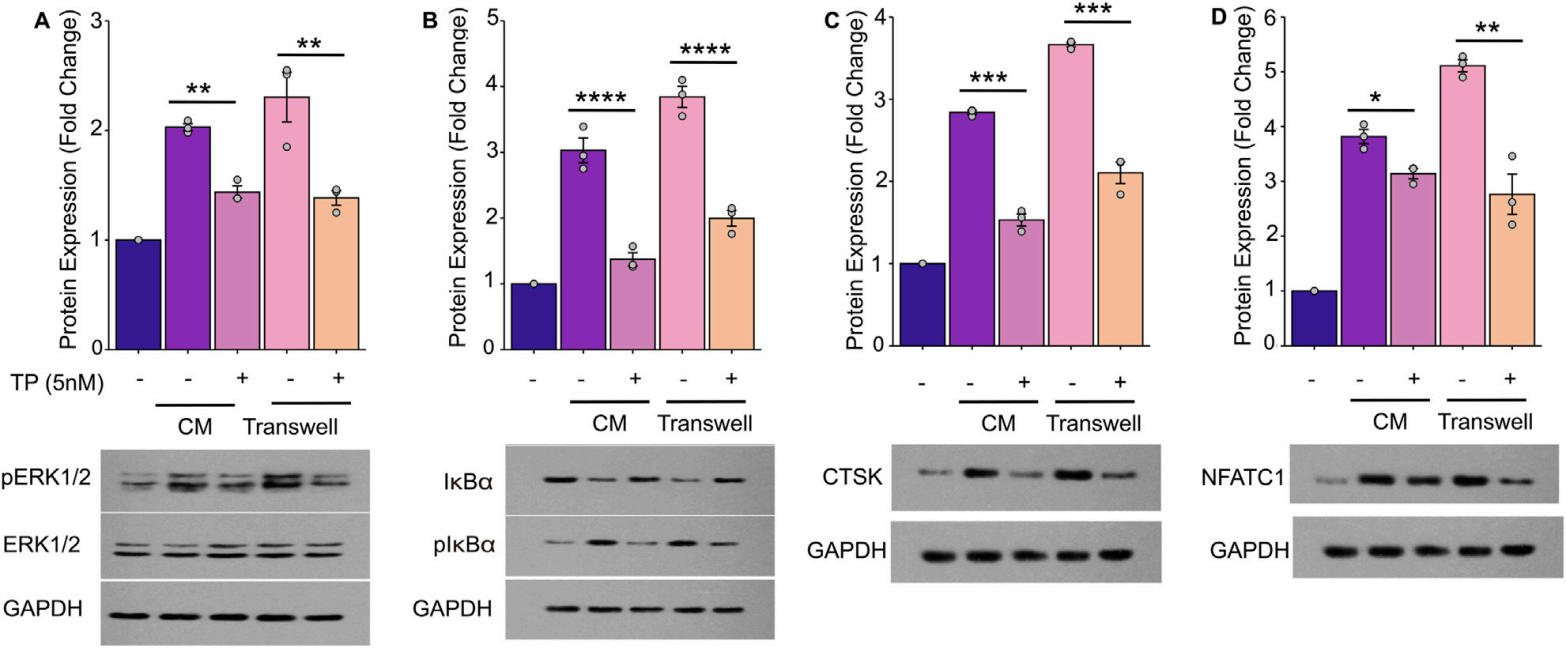
Triptolide Inhibits Breast Cancer Cell-Induced Osteoclastogenesis by Suppressing the NF-κB and ERK Signaling Pathways in RAW264.7 Cells. The RAW264.7 cell lysates were harvested to determine the phosphorylated or nonphosphorylated forms of ERK **(A)**, IκBα **(B)**, CTSK **(C)** and NFATC1 **(D)** by Western blot. **(A)** TP suppresses the phosphorylation of ERK activated by breast cancer cells and blocks the activation of the ERK signaling pathway. **(B)** TP suppresses the phosphorylation of IκBα activated by breast cancer cells and blocks the activation of the NF-κB signaling pathway. **(C,D)** As the ERK and NF-κB signaling pathway is inhibited by TP, the expression of osteoclastogenesis-related markers, such as NFATC1 and CTSK, is decreased. The lower side of each figure shows the bar graph of the expression of p-ERK, p-IκBα, CTSK and NFATC1 by optical density analysis with reference to GAPDH. The results are presented as the mean ± standard error of mean of three independent experiments. *p < 0.05, **p < 0.01, ***p < 0.001. All data were normalized to the NC group.

Prior studies have established that the NF-κB and ERK pathways play crucial roles in osteoclast differentiation and activation (Boyle et al., 2003; Boyce et al., 2015), with TP known to inhibit these pathways in various cellular contexts (Huang et al., 2015; Yanchun et al., 2019). We observed a significant phosphorylation of IκBα, indicative of NF-κB pathway activation in RAW264.7 cells when cultured in the CM for 3 days (an early stage of osteoclast/formations). Additionally, CM-induced phosphorylation of ERK and elevated expression of osteoclast-specific genes such as NFATc1 and CTSK. These findings were further corroborated using a transwell co-culture system, where MC3T3-E1 cells co-cultured with MDA-MB-231 cells similarly activated the NF-κB and ERK pathways, leading to osteoclastogenesis (Figure 4). Further TP treatment significantly reduced the phosphorylation and augmented expression of the effector genes.

### 3.4 Triptolide inhibits RANKL overexpression by osteoblasts in tumor microenvironment

**FIGURE 5:**
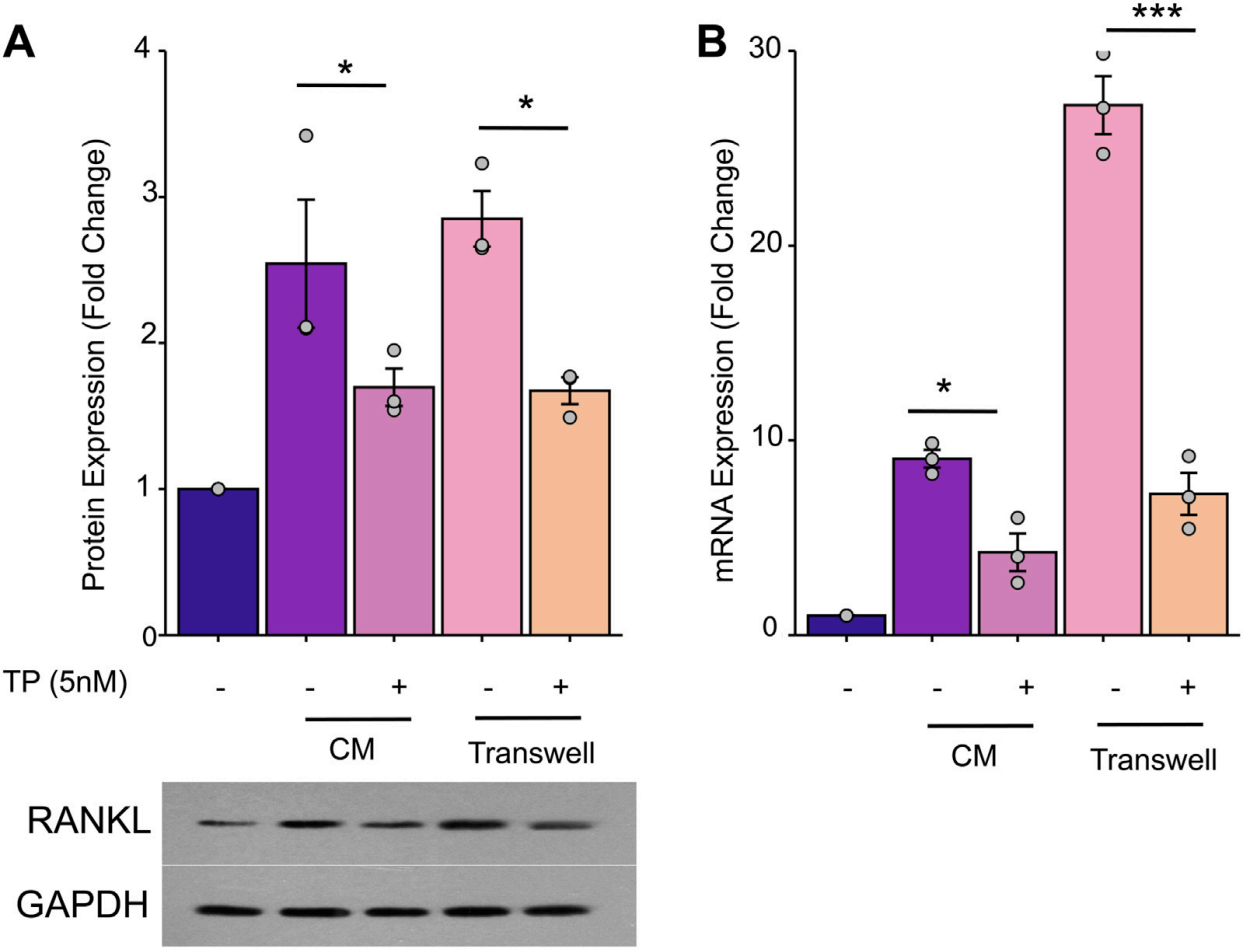
TP inhibits MDA-MB-231 cells-induced overexpression of RANKL in osteoblasts. MC3T3-E1 cells (1*10^6^/well) were incubated with MDA-MB-231 CM (50%CM and 50%-α-MEM) in 6-well plates for 24 h. Or MC3T3-E1 cells (1*10^6^/well) were seeded in the lower chamber and MDA-MB-231 cells (1*10^4^/well) were seeded in the upper chamber of a 6-well transwell plate (0.4um) in a-MEM for 24 h. The MC3T3-El cells lysates were harvested to determine the mRNA or protein by Western blot and RT-PCR. **(A)** TP inhibit RANKL protein overexpession of MC3T3-E1. The bar graph of the expression of RANKL by optical density analysis with reference to GAPDH. **(B)** TP has the same effect on the expression of RANKL mRNA. The results are presented as the mean ± standard error of mean of three independent experiments. *p < 0.05, ***p < 0.001. All data were normalized to the NC group.

Osteoblasts activated by tumor cells secrete significant amounts of RANKL, which indirectly stimulates osteoclastogenesis. To investigate the role of TP in modulating the expression of RANKL by osteoblasts in the tumor microenvironment, mouse osteoblast precursor cells MC3T3-E1 were cultured in CM, with or without TP treatment, and determined the protein and transcriptional expression of RANKL in MC3T3-E1 cells. Our results demonstrated that MC3T3-E1 cells cultured in CM expressed high expression of RANKL. Further, presence of TP significantly inhibited the expression of RANKL levels. Similar results were observed in the presence and absence pf TP when cells were cultured in transwell system (Figure 5). These findings imply that TP can mitigate the overexpression of RANKL in osteoblasts that is induced by breast cancer cells, potentially leading to a decrease in osteoclastogenesis and subsequent bone degradation.

### 3.5 Interaction of PTHrP and Triptolide

**FIGURE 6:**
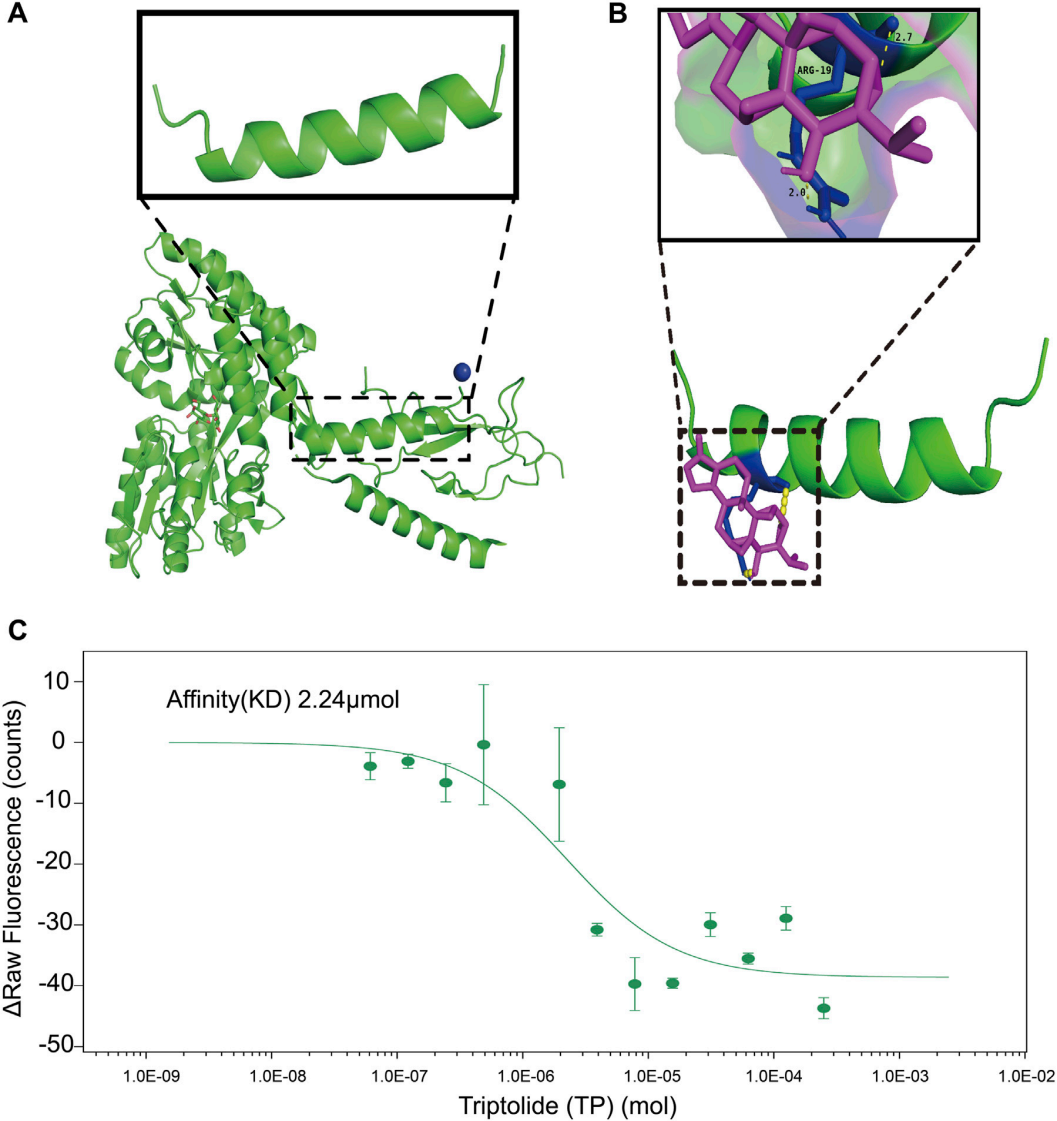
The inhibitory effect of TP on the overexpression of RANKL in osteoblasts within the tumor microenvironment may be attributed to the binding of TP to PTHrP. **(A)** Crystal structure of the extracellular domain of the human parathyroid hormone receptor (PTH1R) in complex with parathyroid hormone-related protein (PTHrP). The enlarged structure is PTHrP fragment. The structure is downloaded from the PDB(3H3G). **(B)** TP binding to PTHrP predicted by AUTODOCK4.2. After 100 calculations, the combined energy between the two is −24.26 kJ/mol. TP and PTHrP have the possibility of combination. The binding site may be the ARG amino acid residue in PTHrP. Hydrogen tendon distance between ligand and amino acid residues is about 2.0–2.7. The green structure is protein. The purple structure is a compound. The yellow indicates hydrogen tendons. The blue indicates bound amino acid residues. **(C)** MST assays on the interaction of TP with the PTHrP (1-34). The affinity (KD) is 2.24 μM. The results are presented three independent experiments. PTHrP sequence: *MQRRLVQQWSVAVFLLSYAVPSCGRSVEGLSRRLKRAVSEHQLLHDKGKSIQDLRRRFFLHHLIAEIHTAEIRATSEVSPNSKPSPNTKNHPVRFGSDDEGRYLTQETNKVETYKEQPLKTPGKKKKGKPGKRKEQEKKKRRTRSAWLDSGVTGSGLEGDHLSDTSTTSLELDSR.*

Molecular Docking is an essential aspect of *in silico* drug development as this technique predicts the interaction between a small molecule (such as nutraceuticals) and a protein at the atomic level. The simulation results demonstrated a binding between TP and PTHrP, with a combined binding energy of −24.26 kJ/mol indicating that TP and PTHrP have a high likelihood of forming a stable complex and likely around the ARG amino acid residue. This residue is important as it potentially forming hydrogen bonds with TP. (Figures 6A,B). The results from MST assay, demonstrated that TP binds to PTHrP(1‐34) with a dissociation constant (Kd) of 2.24 μM (Figure 6C). This is moderate interaction and could potentially influence PTHrP function in biological systems, however, the caution is warranted in extrapolating these findings, and future studies are required to elucidate the underlying mechanisms. While these biophysical and computational approaches provide initial support for a potential interaction, we acknowledge that additional studies, such as PTHrP knockdown or the use of structure–function analogs of TP, would be necessary to fully elucidate the biological relevance of this binding.

## 4 Discussion

Recently, we reviewed the therapeutic potential of Triptolide (TP) as a potential therapeutic target for bone-related disorders (Gang et al., 2022). In this study, we provide the first evidence of the interaction between TP and PTHrP, highlighting its potential therapeutic role in inhibiting the osteoclastogenic effects of PTHrP and its activation of downstream signaling pathways, such as NF-κB and ERK1/2, which promote osteoclast formation. Current therapeutic strategies for bone metastases largely rely on bisphosphonates and denosumab, which effectively target osteoclast-mediated bone resorption. However, these agents do not directly disrupt tumor–bone molecular signaling and are associated with side effects such as osteonecrosis of the jaw and renal impairment (Cahyanur and Pravita, 2024). In contrast, Triptolide not only inhibits osteoclast activity but also modulates upstream tumor-derived factors, such as parathyroid hormone-related protein (PTHrP)-induced RANKL expression, thereby offering a more comprehensive approach to controlling tumor-induced bone destruction. While the clinical application of TP is currently limited by its systemic toxicity, analogs such as Minnelide have shown improved safety profiles in preclinical and early-phase studies, representing a promising direction for future therapeutic development (Propper et al., 2024).

The use of MDA-MB-231 conditioned medium is a widely accepted method to simulate the complex breast cancer tumor microenvironment (Pederson et al., 1999; Patel et al., 2011; Chiou et al., 2021; Dwivedi et al., 2018). Various cytokines released by breast cancer cells (RANKL, IL-1, TNF-α, and M-CSF) directly stimulate the activation of osteoclast. MDA-MB-231 cells do not express RANKL, but this cell line can express TNF-α, M-CSF, IL-1, and other important factors for osteoclastogenesis (Yin et al., 2005; Kang et al., 2005). Even without RANKL, MDA-MB-231 cells or CM cause the changes in the BMCs and also transform the osteoclast precursor cells RAW264.7 and osteoblast precursor cells MC3T3-E1. Previous studies demonstrated that TP inhibits osteoclastogenesis via RANKL-mediated pathways and restores osteoblast differentiation suppressed by TNF-α (Park, 2014; Liu et al., 2017). In contrast, our study uses breast cancer cells as the osteoclastogenic stimulus via both direct co-culture and conditioned media from MDA-MB-231 cells to more accurately model the tumor-bone microenvironment and comprehensively assess TP effects on both tumor–osteoclast and tumor–osteoblast–osteoclast interactions. Though, CM does not isolate the role of individual cytokines but provides a holistic view of tumor-secreted factors which collectively contribute to abnormal osteoclastogenesis. Future studies focusing on the identification and characterization of CM would be worthwhile and intriguing avenue to explore.

PTHrP, is a critical regulator in calcium homeostasis and has been implicated in the development and progression of cancer metastasis, particularly in bone tissues (Zheng et al., 2013; Sanders et al., 2000). The binding of TP to PTHrP could potentially modulate its activity, which might have therapeutic implications, particularly in cancer treatment. The docking simulation results provide a promising indication that TP could interact effectively with PTHrP, potentially leading to significant biological effects. This interaction warrants further investigation in experimental studies to validate the findings and explore the therapeutic potential of this binding. How TP inhibits abnormal RANKL expression in osteoblasts in the tumor microenvironment remains unclear. One possible mechanism could be that TP bind to and sequester PTHrP, thereby preventing its interaction with the PTH1R receptor and downregulating downstream RANKL signaling. This pathway could account for the indirect suppression of osteoclast differentiation. Supporting this hypothesis, previous studies have shown that PTHrP overexpression in breast cancer cells stimulates osteoblast-derived RANKL while suppressing OPG, ultimately enhancing osteoclastogenesis and bone resorption (Kitazawa and Kitazawa, 2002; Kozlow and Guise, 2005). More recently, targeting PTHrP has been shown to reduce osteolytic lesions and osteoclast activity in breast cancer bone metastasis models (Abudourousuli et al., 2022).

**FIGURE 7:**
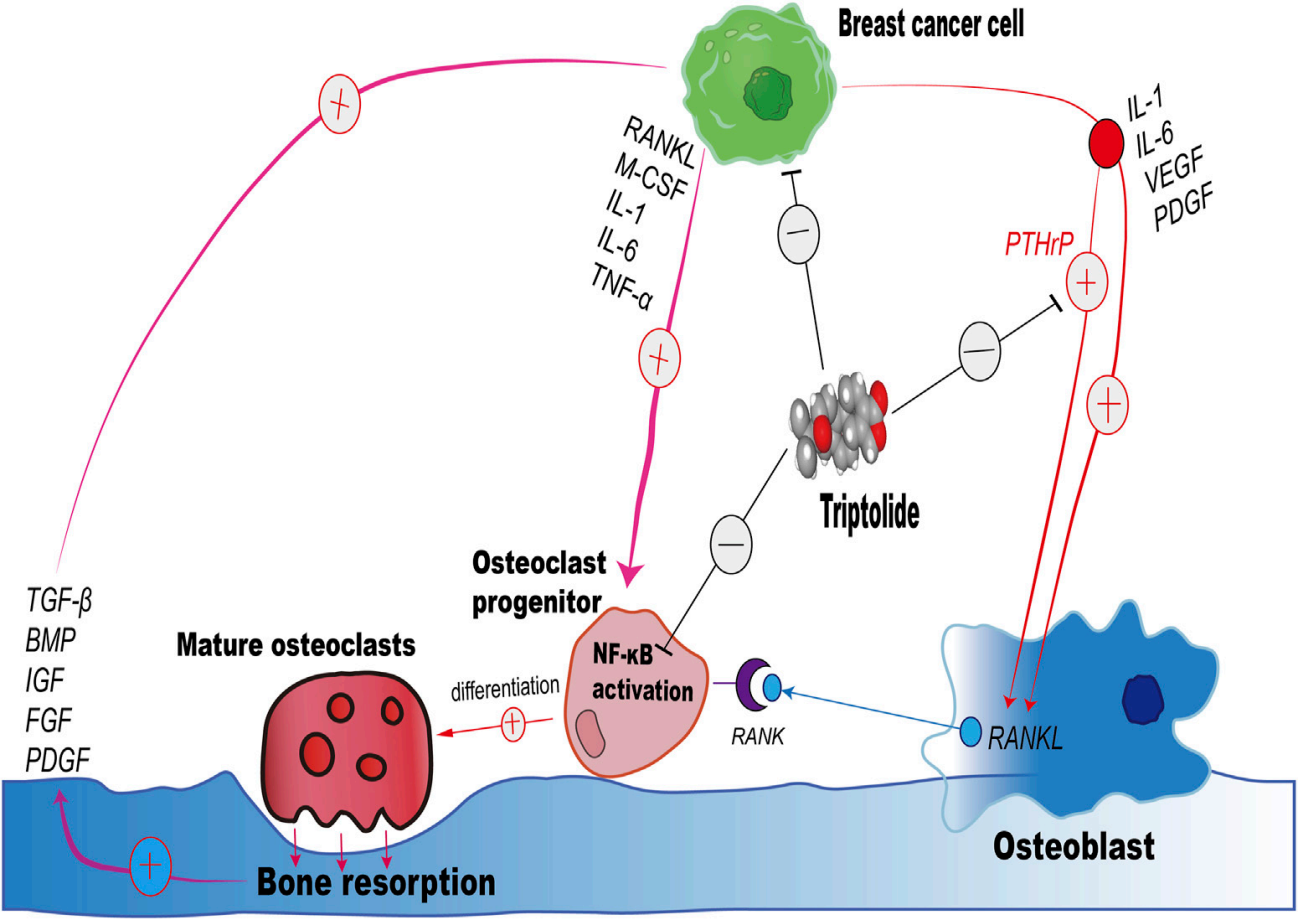
Schematic illustration of the impact of Triptolide on the Interaction Between Breast Cancer Cells, Osteoclasts, and Osteoblasts in Bone Resorption.

In addition to this, several target proteins that have been shown to interact with TP are mostly highly expressed in breast cancer cells, such as XPB, TAB1, ADAM10, DCTPP1, Erα (Noel et al., 2019). Proteins that stimulate the secretion of RANKL by osteoblasts, such as PTHrP, IL-1, TGF-β, and PDGF, inhibit the abnormal RANKL expression by osteoblasts in the tumor microenvironment (Rahman et al., 2015; Fukawa et al., 2022). PTHrP stimulates osteoblasts to express RANKL, which in turn promotes osteoclast formation and bone resorption which further releases growth factors like TGF-β and stimulate cancer growth and the production of osteolytic factors, including PTHrP (Guise and Chirgwin, 2003). Yin et al. have demonstrated that TGF-β, released during bone resorption, enhances the expression of PTHrP by breast cancer cells, which in turn increases RANKL expression by osteoblasts, driving further osteoclastogenesis and bone destruction (Yin et al., 1999). PTHrP is known to promote osteoclastogenesis by enhancing the production of RANKL and by influencing the balance between RANKL and OPG (Osteoprotegerin, a decoy receptor for RANKL) (Boabaid et al., 2004). A significant association of increased serum levels of PTHrP in breast cancer subjects is well documented in literature (Washam et al., 2013; Bucht et al., 1998). Therefore, we speculate that TP may interact with the exogenous protein—PTHrP, blocking the binding of PTHrP to PTH1R on the surface of osteoblasts, inhibiting the excessive secretion of RANKL by osteoblasts in the tumor microenvironment, and indirectly inhibiting osteoclastogenesis, which is one of the effects of TP in the inhibition of bone metastasis (Figure 7). Therefore, we hypothesized that PTHrP might be one of the target proteins of TP, inhibiting the abnormal RANKL expression in osteoblasts in the tumor environment, which served to indirectly inhibit osteoclastogenesis and alleviate the progression of breast cancer bone metastasis.

Triptolide has been shown to reduce osteoclast numbers and protect against titanium particle-induced osteolysis in murine models (Huang et al., 2015), and celastrol significantly inhibited joint erosion and osteoclast differentiation in collagen-induced arthritis mice (Gan et al., 2015). These studies hence support the activity of TP in *in vivo* models. However, a key limitation of this study is the lack of *in vivo* validation of the findings regarding the inhibitory effects of TP on osteoclastogenesis in the breast cancer bone metastasis environment. Although conditioned medium was used to simulate the complex tumor microenvironment, it does not fully replicate the intricate *in vivo* interactions between breast cancer cells, osteoblasts, and osteoclasts. Moreover, the cytotoxicity of TP remains a significant barrier to its clinical application, limiting the therapeutic potential of TP in its current form. While TP showed promising effects *in vitro*, its toxicity profile still remains a significant barrier for its safety and feasibility for long-term use.

Our *in vitro* observations are corroborated by previous *in vivo* research demonstrating that TP and its derivatives inhibit osteoclast activity and bone resorption in various disease contexts. Triptolide was shown to reduce osteoclast numbers and protect against titanium particle-induced osteolysis in murine models (Huang et al., 2015), and celastrol significantly inhibited joint erosion and osteoclast differentiation in collagen-induced arthritis mice (Gan et al., 2015). However, future studies should utilize animal models of breast cancer-induced bone metastasis to assess the *in vivo* efficacy of Triptolide. Such studies would enable validation of its observed anti-resorptive effects and allow for a comprehensive assessment of pharmacokinetics, bioavailability, and systemic toxicity.

Despite its potent anti-inflammatory and anticancer properties, TP remains in the experimental research stage, with the known challenges such as a narrow therapeutic window and systemic toxicity, particularly hepatotoxicity, nephrotoxicity, and reproductive toxicity under prolonged exposure (Bao and Dai, 2011). These safety concerns have historically limited its direct clinical application. However, ongoing research has focused on developing structurally related analogs and novel delivery systems—such as nanoparticle encapsulation, prodrugs, and antibody-drug conjugates—to improve its selectivity for cancer cells while reducing systemic exposure and toxicity (Liu et al., 2021). However, there is ample evidence in the literature to demonstrate the beneficial effect of TP on cancer and orthopedic diseases. In addition, comparative studies evaluating the relative efficacy and safety of TP in comparison with clinically used agents such as bisphosphonates would be valuable. Based on chemical structure of TP, many TP derivatives, such as Minnelide, are now available, which act similarly to TP but are less biotoxic and have been proposed as a potential candidate for clinical trials (Banerjee and Saluja, 2015; Chugh et al., 2012). Additionally, future research should delve deeper into the molecular mechanisms by which TP modulates the PTHrP/RANKL/OPG axis and its downstream signaling pathways. This could include exploring the effects of TP on other signaling pathways involved in osteoclastogenesis, such as the NF-κB and ERK1/2 pathways, and determining whether TP can be combined with other therapeutic agents to enhance its efficacy.

## Data Availability

The original contributions presented in the study are included in the article/Supplementary Material, further inquiries can be directed to the corresponding author.
