# Supplementary material for: Therapeutic potential of Triptolide in inhibiting breast cancer-induced bone destruction – PTHrP as a therapeutic target
Source: Front Pharmacol. 2025 May 15;16:1512631. doi: 10.3389/fphar.2025.1512631 (PMC12119609; doi:10.3389/fphar.2025.1512631)
Supplement: Supplementary file 1 [file Table1.docx]

Supplementary Material

# Supplementary Reagents Data

| reagents | Cat No | Company |
| --- | --- | --- |
| Dulbecco’s Modified Eagle Medium (DMEM) | 11965-092 | GIBCO (America) |
| α-Minimum Essential Medium(αMEM) | 12571-063 | GIBCO (America) |
| Receptor Activator of Nuclear Factor-κ B Ligand(RANKL) | 315-11C | PeproTech (America) |
| Parathyroid hormone-related protein(PTHrP) | Ag24533 | Proteintech(Wuhan,China) |
| Fetal bovine serum(FBS) | B118-500 | Nobimpex (South America) |
| Triptolide(TP) | B20709 | Shanghai yuanye Bio-Technology Co. Ltd (Shanghai, China) |
| CCK-8 reagent | G4103 | Servicebio (Wuhan,China) |
| Trizol reagent | G3013 | Servicebio (Wuhan,China) |
| Phenylmethanesulfonyl fluoride(PMSF) | G2008 | Servicebio (Wuhan,China) |
| phosphatase inhibitors | G2007 | Servicebio (Wuhan,China) |
| RT-PCR reagents | CW2020M, CW3888M | Cwbiotech (Jiangsu,China) |
| Radioimmunoprecipitation(RIPA) | P0013B | Beyotime(Shanghai,China) |
| Antibody Erk1/2 | P20704 | Promab(Hunan，China) |
| Antibody P-ERK1/2 | P20216 | Promab(Hunan，China) |
| Antibody CTSK | P34752 | Promab(Hunan，China) |
| Antibody NFATC1 | P23529 | Promab(Hunan，China) |
| Antibody IKBα | P20973 | Promab(Hunan，China) |
| Antibody P-IKBα | P20122 | Promab(Hunan，China) |
| Antibody RANKL | P13117 | Promab(Hunan，China) |
| Antibody GAPDH | 60004-1-Ig | Proteintech(Wuhan,China) |
| Antibody Goat anti-mouse | SA00001-1 | Proteintech(Wuhan,China) |
| Antibody Goat anti-rabbit | SA00001-2 | Proteintech(Wuhan,China) |
| RED-tris-NTA 2nd | Cat#MO-L018 | Nanotemper(Germany) |
| Capillaries | Cat#MO-K022-1000 Count | Nanotemper(Germany) |
